# Single-Session of Combined tDCS-TMS May Increase Therapeutic Effects in Subjects With Tinnitus

**DOI:** 10.3389/fneur.2020.00160

**Published:** 2020-03-27

**Authors:** Eun Bit Bae, Jun Ho Lee, Jae-Jin Song

**Affiliations:** ^1^Interdisciplimentary Program in Neuroscience, Seoul National University, Seoul, South Korea; ^2^Laboratory of Electrophysiology, Department of Otorhinolaryngology, Center of Medical Research Innovation, Seoul National University Hospital, Seoul, South Korea; ^3^Department of Otorhinolaryngology-Head and Neck Surgery, Seoul National University Bundang Hospital, Seongnam-si, South Korea; ^4^Department of Otorhinolaryngology-Head and Neck Surgery, Seoul National University College of Medicine, Seoul National University Hospital, Seoul, South Korea

**Keywords:** tinnitus, transcranial direct current stimulation, transcranial magnetic stimulation, neuromodulation, tinnitus handicap inventory, tinnitus intensity, tinnitus distress, tinnitus perception

## Abstract

To treat motor and psychiatric disorders, transcranial direct current stimulation (tDCS) and transcranial magnetic stimulation (TMS) are used in clinics worldwide. We combined these two types of neuromodulation technique to increase the effective response of a single session of neuromodulation in subjective tinnitus. Eighty tinnitus subjects were split into four different treatment groups: tDCS, tDCS with sham TMS, tDCS-TMS, and TMS group. Subjects were given 1.5 mA tDCS on the bi-frontal area and TMS stimulated the contralateral single side of the temporo-parietal cortex with 200 pulses at 1 Hz stimulation. Comparing pre-treatment questionnaire scores to post-treatment questionnaire scores, all four groups showed statistically significant improvements. Although there was no significant difference among group comparison, the largest mean difference was shown in the combined group, especially for tinnitus intensity and tinnitus-related distress. Responders in the combined group were the highest for VAS intensity, with a maximum of 80% of twenty subjects. To summarize, dual-neuromodulation responders could consist of responders of frontal tDCS and temporal TMS. In addition, abnormal activity in the frontal or temporal area of the responders is presumed to be modulated by treatment and will be suggested as the target areas in future studies.

## Introduction

Regardless of age and gender, tinnitus can be developed at any point from childhood onwards. Hearing loss can cause hyperactivity in the bottom-up hearing pathway from the peripheral cochlear nerve to the auditory cortex (1–6). Maladapted signals feed back to the cortex from damaged hair cells or the cochlear nerve. Also, this process may cause central gain enhancement which can be detected as hyperactivity outside of the brain via neuroimaging techniques (7, 8). Previous studies have identified that tinnitus-related cortical circuits are associated with cognition, memory, and emotion (7–11). Some studies also discovered via functional neuroimaging that tinnitus can change these circuits into a strong maladapted connection (12–14). Recent tinnitus-related study results support this concept of a central mechanism that had already been discussed some decades ago (15, 16).

Transcranial magnetic stimulation (TMS) and transcranial direct current stimulation (tDCS) have been used world-wide for non-invasive treatments via stimulation outside of the skull. The treatment modulates neuronal activity and this neuronal modulation then has therapeutic effects on psychiatric and neuro-muscular disorders (17–20). TMS has been approved for the treatment of depression and stroke by the US Food and Drug Administration, and tDCS has also been approved for depression and peripheral motor disorders by Conformité Européenne (CE) (21). Expecting similar therapeutic effects, TMS has begun to be used in clinical trials for tinnitus treatment (22, 23). In this way, by applying an effective method for diseases other than tinnitus, results indicating therapeutic effects of tinnitus have continuously been reported.

Response to a single session of frontal tDCS was reported to be 22.1–29% in previous studies (24, 25). On the other hand, temporal tDCS showed a response rate of 6.9% at 1.5 mA and 17.4% at 2.0 mA (26). In an 8-session study of frontal tDCS, 39% of the study subjects were found to be responders (27). As for TMS, previous studies have reported that single session TMS was effective in 15–23.9% of the participants with regard to the improvement of tinnitus (28, 29). The same research group reported that low frequency TMS was more effective than high frequency TMS, with low frequency TMS showing a response rate of 25% (30). Responders increased in repetitive TMS (rTMS) studies, i.e., rTMS at one area showed a response rate of 39 – 43 %, while rTMS at two different areas showed a rate of 43–48% (28, 31, 32).

Overall, previous tDCS- or TMS studies for the treatment of tinnitus have shown that responders reporting positive outcomes were, at a maximum, around 50% of the participants (23, 25–28, 31, 32). There has been no previous study in which all participants have experienced a treatment effect because of different study designs and heterogenous patient groups, and a standard neuromodulation protocol for tinnitus has not been established yet. In this regard, the current study compares four different study groups with various combinations of single-session tDCS and TMS.

## Materials and Methods

### Subjects

Eighty-four subjects (an age range from 25 to 73 years) with subjective tinnitus were enrolled and participated in this clinical trial from August 2016 to July 2018. Of them, four subjects were excluded. Patients who had serious neurological disorders, severe psychiatric disorders or schizophrenia, and patients whose main complication was not subjective tinnitus, such as pulsatile tinnitus and Meniere's disease, were excluded from the study.

### Clinical Trial

The aim of the study was to evaluate the effectiveness of a single session of combined tDCS and TMS treatment on subjective tinnitus as compared with single treatment groups. The clinical trial was approved by the Institutional Review Board of the Seoul National University, Bundang Hospital on August 29, 2016 (IRB No.: B-1607-355-004) and the clinical trial followed the guidelines of the Declaration of Helsinki. Also, the clinical trial has been registered at ClinicalTrials.gov (ID: NCT04262050). All included patients gave their written informed consent. Research volunteers who agreed to participate in the clinical trial were gathered from the tinnitus clinic of the Department of Otorhinolaryngology Head-and-Neck Surgery, Seoul National University, Bundang Hospital.

Subjects were randomly allocated to one of four types of treatments to receive a clinical consult, except for two subjects who had to be assigned to the tDCS group because of their history of coronary artery stenting. Subjects in both the combined tDCS and TMS group, and the tDCS with sham TMS group were given the same information about the treatment stimulation procedures. The total number of subjects was 80 with four groups of 20 each, and the male to female ratio was nearly 1:1 in all four experimental groups (Table 1). The clinical characteristics of the subjects in the four groups were not statistically significant [ANOVA, pre-treatment THI (*P* = 0.838), VAS intensity (*P* = 0.613), VAS distress (*P* = 0.517), VAS perception (*P* = 0.853), age (*P* = 0.478), tinnitus durations (*P* = 0.213), and gender ratio (*P* = 0.849)].

**Table 1:** Demographic characteristics of the study subjects in all four groups.

				**Pre-treatment questionnaire score**			
**Group**	**Age**	**Duration**	**M:F**	**THI**	**Intensity**	**Distress**	**Perception**
				**Mean ± SD**	**Mean ± SD**	**Mean ± SD**	**Mean ± SD**
tDCS	53.8 ± 13.8	3.4 ± 5.0	11:09	55.7 ± 21.0	6.5 ± 1.9	6.8 ± 2.2	77.3 ± 24.0
tDCS-shTMS	54.4 ± 12.9	3.4 ± 5.4	9:11	54.3 ± 19.5	6.7 ± 2.0	7.1 ± 1.7	75.0 ± 27.0
tDCS-TMS	57.5 ± 8.4	7.7 ± 11.1	9:11	58.6 ± 19.0	7.3 ± 2.0	7.6 ± 2.0	81.0 ± 22.7
TMS	51.5 ± 13.0	4.1 ± 6.8	11:09	53.0 ± 21.2	6.6 ± 2.2	6.6 ± 2.4	80.5 ± 25.0
*P*-value	0.604	0.316	0.849	0.799	0.439	0.485	0.876

*THI, tinnitus handicap inventory; M, male; F, female; SD, standard deviation; Tx, treatment; tDCS, transcranial direct current stimulation; TMS, transcranial magnetic stimulation; sh, sham*.

### Treatments

Experimental groups consisted of four different combinations of transcranial stimulations: (1) the tDCS group, (2) tDCS with sham TMS (tDCS-shTMS) group, (3) combined tDCS and TMS (tDCS-TMS) group, and (4) TMS group. According to previous studies, bifrontal tDCS was used in the current study as it is more effective than temporal tDCS with regard to tinnitus control (24). Using TMS, we stimulated the contralateral temporal area of the tinnitus side.

Subjects who were assigned to the tDCS (DC-stimulator Plus, Neuroconn, Germany) group were given a 1.5 mA-DC stimulation on both frontal areas for 20 min; the anode was placed on the left frontal area (F3), and the cathode on the right frontal area (F4) (33–35). While a tingling or stinging-like sensations were a commonly predictable response for about the first 3–5 min, none of the subjects who underwent tDCS asked us to stop the stimulation.

Subjects who were assigned to the TMS groups (the tDCS-TMS group and the TMS group) had their resting-state motor thresholds (RMT) measured by the MagPro X100 (Tonica Elecktronik A/S, Denmark) and were given a stimulation at 80% intensity of the measured RMT, which ranged from 5% to a maximum of 30% stimulator output (29, 36). The RMT was defined as the minimum stimulus intensity. The response was defined as the minimum stimulus intensity, which was reproducible by 3 times at about 50 μV. Using the 10–20 system, a single session of TMS was applied to the contralateral temporo-parietal cortex of the subject's tinnitus side, between T3 or T4 and the P3 or P4, for 3 min 20 s with 200 pulses at a low frequency of 1Hz (29, 30, 36, 37). The recording electrode was placed on the skin over the Abdoctor Pollicis Brevis muscle, and the reference electrodes were positioned to the interphalangeal joint. A ground electrode was applied around the flexor carpi radialis muscle.

Considering the placebo effects of the tDCS-TMS group, we did not inform the tDCS-shTMS group and tDCS-TMS group of the differences between the two groups. The subjects included in the tDCS-shTMS group had their RMT measured, and a figure-of-eight coil was placed on the temporal area of the contralateral side of tinnitus. The coil was set up on the temporal area with the stimulus facing outward.

**Figure 1:**
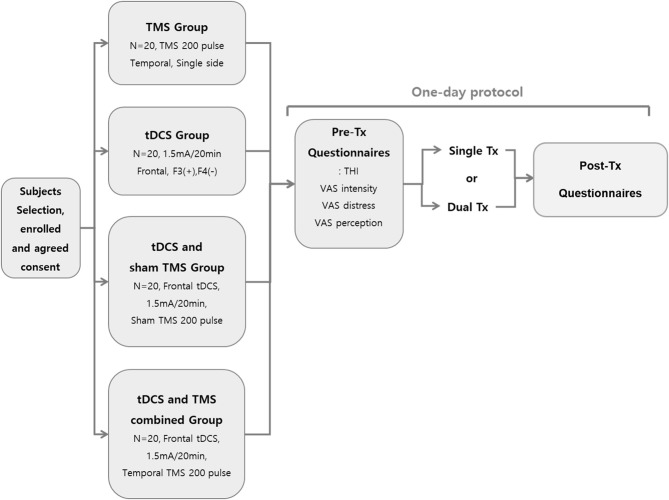
Schematic summary of the study protocol.

Figure 1 summarizes the study protocol. On the second visit day, after the first day of treatment, two subjects complained of headaches lasting 2 to 3 h. Of them, one received tDCS and the other received tDCS with TMS.

### Evaluation of Treatment Effects

The therapeutic effect of each treatment group was assessed using four questionnaires: tinnitus handicap inventory (THI), visual analog scales (VAS) of tinnitus intensity (loudness), distress (annoyance), and perception (awareness) (38, 39). Subjects completed the four questionnaires both before and after treatment.

### Data Analysis

**Figure 2:**
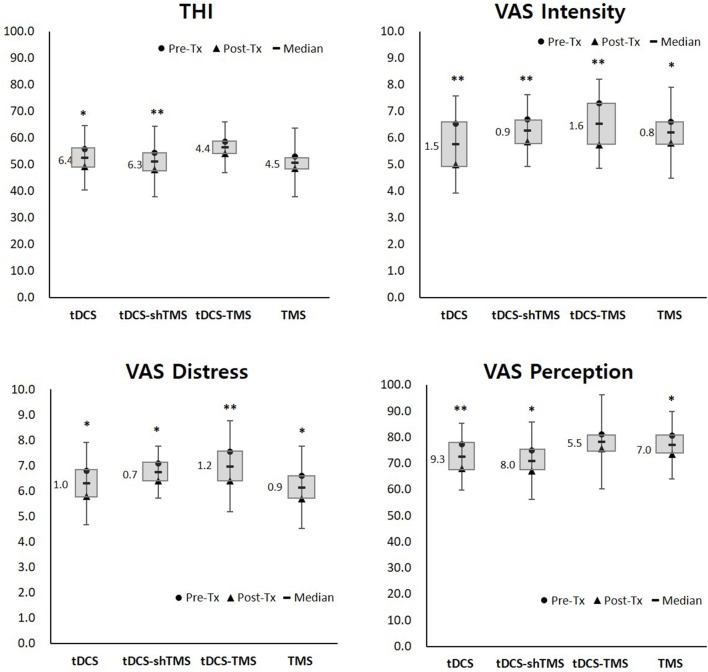
Pre- and post-treatment questionnaire scores of the four study groups. Single asterisk (*) designates *P* < 0.05 while double asterisks (**) designate *P* < 0.01. THI, tinnitus handicap inventory; VAS, visual analog scale; Tx, treatment; tDCS, transcranial direct current stimulation; TMS, transcranial magnetic stimulation; sh, sham.

**Figure 3:**
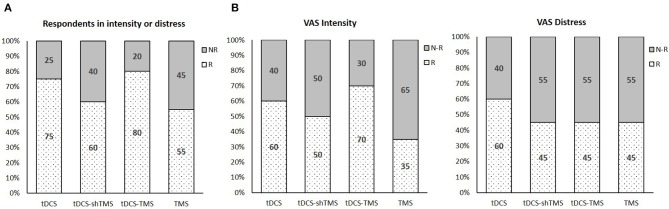
The percentage neuromodulation responders and non-responders with regard to respondents in intensity or distress **(A)**, VAS intensity **(B)**, and VAS distress **(C)** in the 4 study groups. VAS, visual analog scale; Tx, treatment; tDCS, transcranial direct current stimulation; TMS, transcranial magnetic stimulation; sh, sham.

Each pre- and post-treatment questionnaire score was analyzed within a treatment group via Wilcoxon signed ranks test analysis. The mean values of the pre- and post-treatment scores of each group were obtained to confirm the differences, as shown in the box of Figure 2. Also, comparisons of the four questionnaire scores among treatment groups were performed via Kruskal–Wallis test (Figure 3).

The criterion for defining whether a subject is a respondent of a questionnaire is set by the minimum response scores. We set the responder criterion with regard to the THI and VAS perception as a decrease of a score of 5 or more, and the criterion for the VAS intensity and distress as a decrease of a score of 0.5 after treatment. Between and within group comparisons were also done for treatment responders. Pre- and post-treatment scores were analyzed by two related tests for comparing within groups, and a median test was done for between group comparisons. All the statistical analysis presented in the current study were performed by SPSS v.23 (IBM SPSS Statistics for Windows, Armonk, USA).

## Results

### Pre-post Treatment Score Comparisons

Pre-treatment THI scores ranged from 12 to 92, and the mean pre-treatment THI scores of the four groups were designated as having moderate (THI score of 38–56) to severe (58–76) tinnitus (40) (Table 1). Figure 2 and Table 2 shows the results of the pre- and post-treatment differences within each group. With regard to the THI score, the tDCS and tDCS-shTMS groups showed statistically significant improvements (tDCS, *P* = 0.030; tDCS-shTMS, *P* = 0.047). VAS intensity and distress showed significant improvements (*P* < 0.05^*^) in all four groups. For tinnitus perception, the tDCS, tDCS-shTMS, and TMS groups showed significant improvements (*P* = 0.004, *P* = 0.025, and *P* = 0.026, respectively), but the tDCS-TMS group did not (*P* = 0.186) (Figure 2). In all four groups, responders and non-responders showed no statistically significant differences with regard to demographic factors such as age, duration of tinnitus, perception, gender, or questionnaire scores.

**Table 2:** The responders with regard to either VAS intensity or distress.

	**Responders**					
**Group**	**THI**	**Intensity**	**Distress**	**Perception**	**Intensity or distress**	
	***P*-value**	***P*-value**	***P*-value**	***P*-value**	**R**	**NR**
tDCS	0.027	0.002	0.01	0.008	15	5
tDCS-shTMS	0.028	0.008	0.007	0.026	12	8
tDCS-TMS	0.032	0.001	0.007	0.223	16	4
TMS	0.109	0.017	0.006	0.027	11	9
Total N	80	80	80	80	54	26

*THI, tinnitus handicap inventory; R, responder; NR, non-responder; tDCS, transcranial direct current stimulation; TMS, transcranial magnetic stimulation; sh, sham*.

### The Percentage of Responders in All 4 Groups

Figure 3A shows the percentage of responders in all four groups with regard to either VAS intensity or VAS distress. As shown in Figure 3A, the tDCS-TMS group showed the highest percentage of responders (80%) with regard to either VAS intensity or VAS distress as compared to the other three groups. When we only considered the VAS intensity, the tDCS-TMS group indicated the highest percentage of responders (70%) (Figure 3B). Meanwhile, with regard to the VAS distress, the tDCS group showed the highest percentage of responders (60%) (Figure 3C).

## Discussion

In this study, all four groups showed statistically significant improvement with regard to VAS intensity and distress. Although <50% of the subjects responded in the tDCS-shTMS, tDCS-TMS, and TMS groups with regard to VAS distress, significant improvements were achieved in all four groups owing to some responders with stark improvements after treatment.

Of the 25 THI questions, most consist of questions about daily social lives, for example, “Does your tinnitus make it difficult for you to enjoy life?,” while VAS perception asks for the average percentage of time spent actively noticing tinnitus during the daytime of a routine day. Because the current study design was a single session neuromodulation and the review time was as short as 5 min, it was difficult to reflect the immediate treatment effect with regard to THI or VAS perception scores. In this regard, of the four questionnaire scores used in the current study, VAS intensity and distress scores may have reflected the immediate treatment effect better than THI or VAS perception scores. Although it was not statistically significant, the mean improvements after treatment in VAS intensity and distress in the tDCS-TMS group were larger than those of the other three groups (Figure 2).

Also, the tDCS-TMS group showed the highest percentage of responders (80%) with regard to either VAS intensity or VAS distress compared to the other three groups (Figure 3A). With regard only to the VAS intensity, the tDCS-TMS group indicated the highest percentage of responders (70%) (Figure 3B).

Commonly involved areas reported in most tinnitus-related functional imaging studies are the dorsal lateral prefrontal cortex, tempo-parietal areas, and amygdala (10, 11, 41, 42). These areas have shown relatively increased activity in tinnitus subjects as compared with normal controls in various functional neuroimaging studies using fMRI, PET, and EEG (7, 8, 13, 43, 44). Based on these previous studies, most of the previous tDCS studies on tinnitus have utilized bifrontal tDCS. Also, we have utilized tDCS using a right anode and left cathode because a previous study has reported that no tinnitus-suppressing effect was found for tDCS with s left anode and right cathode, while tDCS with right anode and left cathode modulated tinnitus perception in 29.9% of the study subjects (24). Our results have shown that left anodal stimulation, which has been proven to be effective for depression (33–35), is effective in abating tinnitus.

The current study has several limitations. First, because both tDCS and TMS are known to be effective in improving depression, significant tinnitus improvements in the current study might have partially been affected by mere improvement of depression, not tinnitus itself. Future studies exploring changes both in tinnitus-related questionnaire scores and depression-related questions such as The Hospital Anxiety and Depression Scale (45) should be performed. Second, although we have found that tDCS-TMS has the potential to improve the neuromodulatory effect, thereby creating an improvement of tinnitus, the results are limited as we have utilized only 200 pulses of low-frequency TMS due to time and space constraints in our hospital. Most of the previous tinnitus studies utilizing low- frequency TMS used 2,000 stimuli (46–48). In this regard, future tDCS-TMS studies using conventional TMS with 2,000 stimuli should be performed to explore further additive neuromodulatory effects on tinnitus.

## Conclusion

Taken together, the current study showed a tendency of additive effect in the tDCS-TMS group when compared with tDCS-only or TMS-only groups with regard to the treatment of tinnitus. Because 200-pulse-TMS with combined tDCS did not show significant differences when compared with the other three groups, future studies utilizing increased pulse numbers of TMS are warranted.

## Data Availability Statement

The datasets generated for this study are available on request to the corresponding author.

## Ethics Statement

The studies involving human participants were reviewed and approved by Institutional Review Board of the Seoul National University Bundang Hospital. The patients/participants provided their written informed consent to participate in this study.

## Author Contributions

EB and J-JS designed the study. EB performed the overall procedure, obtained the data, analyzed, and derived the results under the J-JS's supervision. JL reviewed the manuscript.

### Conflict of Interest

The authors declare that the research was conducted in the absence of any commercial or financial relationships that could be construed as a potential conflict of interest.

## References

[1] Pilati N Ison MJ Barker M Mulheran M Large CH Forsythe ID. Mechanisms contributing to central excitability changes during hearing loss. Proc Natl Acad Sci USA. (2012) 109:8292–7. 10.1073/pnas.1116981109 22566618 PMC3361412

[2] Sun W Deng A Jayaram A Gibson B. Noise exposure enhances auditory cortex responses related to hyperacusis behavior. Brain Res. (2012) 1485:108–16. 10.1016/j.brainres.2012.02.008 22402030

[3] Hebert S Fournier P Norena A. The auditory sensitivity is increased in tinnitus ears. J Neurosci. (2013) 33:2356–64. 10.1523/JNEUROSCI.3461-12.2013 23392665 PMC6619157

[4] Auerbach BD Rodrigues PV Salvi RJ. Central gain control in tinnitus and hyperacusis. Front Neurol. (2014) 5:206. 10.3389/fneur.2014.00206 25386157 PMC4208401

[5] Kim SH Jang JH Lee SY Han JJ Koo JW Vanneste S. Neural substrates predicting short-term improvement of tinnitus loudness and distress after modified tinnitus retraining therapy. Sci Rep. (2016) 6:29140. 10.1038/srep29140 27381994 PMC4933976

[6] Lee SY Nam DW Koo JW De Ridder D Vanneste S Song JJ. No auditory experience, no tinnitus: Lessons from subjects with congenital- and acquired single-sided deafness. Hear Res. (2017) 354:9–15. 10.1016/j.heares.2017.08.002 28826043

[7] Vanneste S Joos K Langguth B To WT De Ridder D. Neuronal correlates of maladaptive coping: an EEG-study in tinnitus patients. PLoS ONE. (2014) 9:e88253. 10.1371/journal.pone.0088253 24558383 PMC3928191

[8] Vanneste S Van De Heyning P De Ridder D. Tinnitus: a large VBM-EEG correlational study. PLoS ONE. (2015) 10:e0115122. 10.1371/journal.pone.0115122 25781934 PMC4364116

[9] Leaver AM Turesky TK Seydell-Greenwald A Morgan S Kim HJ Rauschecker JP. Intrinsic network activity in tinnitus investigated using functional MRI. Hum Brain Mapp. (2016) 37:2717–35. 10.1002/hbm.23204 27091485 PMC4945432

[10] Shore SE Roberts LE Langguth B. Maladaptive plasticity in tinnitus–triggers, mechanisms and treatment. Nat Rev Neurol. (2016) 12:150–60. 10.1038/nrneurol.2016.12 26868680 PMC4895692

[11] Vanneste S Song JJ De Ridder D. Thalamocortical dysrhythmia detected by machine learning. Nat Commun. (2018) 9:1103. 10.1038/s41467-018-02820-0 29549239 PMC5856824

[12] De Ridder D Vanneste S Weisz N Londero A Schlee W Elgoyhen AB. An integrative model of auditory phantom perception: tinnitus as a unified percept of interacting separable subnetworks. Neurosci Biobehav Rev. (2014) 44:16–32. 10.1016/j.neubiorev.2013.03.021 23597755

[13] Chen YC Li X Liu L Wang J Lu CQ Yang M. Tinnitus and hyperacusis involve hyperactivity and enhanced connectivity in auditory-limbic-arousal-cerebellar network. Elife. (2015) 4:e06576. 10.7554/eLife.06576.012 25962854 PMC4426664

[14] Chen YC Chen H Bo F Xu JJ Deng Y Lv H. Tinnitus distress is associated with enhanced resting-state functional connectivity within the default mode network. Neuropsychiatr Dis Treat. (2018) 14:1919–27. 10.2147/NDT.S164619 30122924 PMC6078076

[15] Valergakis FE. Radio-isotope brain scanning in otological practice. Laryngoscope. (1967) 77:2178–88. 10.1288/00005537-196712000-00008 6065535

[16] Shulman A Seitz MR. Central tinnitus–diagnosis and treatment. Observations simultaneous binaural auditory brain responses with monaural stimulation in the tinnitus patient. Laryngoscope. (1981) 91:2025–35. 10.1288/00005537-198112000-00005 7321723

[17] Takeuchi N Tada T Matsuo Y Ikoma K. Low-frequency repetitive TMS plus anodal transcranial DCS prevents transient decline in bimanual movement induced by contralesional inhibitory rTMS after stroke. Neurorehabil Neural Repair. (2012) 26:988–98. 10.1177/1545968311433295 22412170

[18] Lefebvre S Dricot L Laloux P Gradkowski W Desfontaines P Evrard F. Neural substrates underlying stimulation-enhanced motor skill learning after stroke. Brain. (2015) 138(Pt 1):149–63. 10.1093/brain/awu336 25488186 PMC4441084

[19] Torres F Villalon E Poblete P Moraga-Amaro R Linsambarth S Riquelme R. Retrospective evaluation of deep transcranial magnetic stimulation as add-on treatment for Parkinson's Disease. Front Neurol. (2015) 6:210. 10.3389/fneur.2015.00210 26579065 PMC4620693

[20] Liu A Bryant A Jefferson A Friedman D Minhas P Barnard S. Exploring the efficacy of a 5-day course of transcranial direct current stimulation (TDCS) on depression and memory function in patients with well-controlled temporal lobe epilepsy. Epilepsy Behav. (2016) 55:11–20. 10.1016/j.yebeh.2015.10.032 26720704

[21] Fregni F Nitsche MA Loo CK Brunoni AR Marangolo P Leite J. Regulatory considerations for the clinical and research use of Transcranial Direct Current Stimulation (tDCS): review and recommendations from an expert panel. Clin Res Regul Aff. (2015) 32:22–35. 10.3109/10601333.2015.980944 25983531 PMC4431691

[22] De Ridder D De Mulder G Walsh V Muggleton N Sunaert S Moller A. Magnetic and electrical stimulation of the auditory cortex for intractable tinnitus. Case report J Neurosurg. (2004) 100:560–4. 10.3171/jns.2004.100.3.0560 15035296

[23] Fregni F Marcondes R Boggio PS Marcolin MA Rigonatti SP Sanchez TG. Transient tinnitus suppression induced by repetitive transcranial magnetic stimulation and transcranial direct current stimulation. Eur J Neurol. (2006) 13:996–1001. 10.1111/j.1468-1331.2006.01414.x 16930367

[24] Vanneste S Plazier M Ost J van der Loo E Van de Heyning P De Ridder D. Bilateral dorsolateral prefrontal cortex modulation for tinnitus by transcranial direct current stimulation: a preliminary clinical study. Exp Brain Res. (2010) 202:779–85. 10.1007/s00221-010-2183-9 20186404

[25] De Ridder D Vanneste S. EEG Driven tDCS Versus Bifrontal tDCS for Tinnitus. Front Psychiatry. (2012) 3:84. 10.3389/fpsyt.2012.00084 23055986 PMC3457073

[26] Joos K De Ridder D Van de Heyning P Vanneste S. Polarity specific suppression effects of transcranial direct current stimulation for tinnitus. Neural Plast. (2014) 2014:930860. 10.1155/2014/930860 24812586 PMC4000666

[27] Rabau S Shekhawat GS Aboseria M Griepp D Van Rompaey V Bikson M. Comparison of the long-term effect of positioning the cathode in tDCS in tinnitus patients. Front Aging Neurosci. (2017) 9:217. 10.3389/fnagi.2017.00217 28804455 PMC5532430

[28] Vanneste S De Ridder D. Differences between a single session and repeated sessions of 1 Hz TMS by double-cone coil prefrontal stimulation for the improvement of tinnitus. Brain Stimul. (2013) 6:155–9. 10.1016/j.brs.2012.03.019 22658239

[29] Schecklmann M Lehner A Gollmitzer J Schmidt E Schlee W Langguth B. Repetitive transcranial magnetic stimulation induces oscillatory power changes in chronic tinnitus. Front Cell Neurosci. (2015) 9:421. 10.3389/fncel.2015.00421 26557055 PMC4617176

[30] De Ridder D Song JJ Vanneste S. Frontal cortex TMS for tinnitus. Brain Stimul. (2013) 6:355–62. 10.1016/j.brs.2012.07.002 22853891

[31] Mennemeier M Chelette KC Allen S Bartel TB Triggs W Kimbrell T. Variable changes in PET activity before and after rTMS treatment for tinnitus. Laryngoscope. (2011) 121:815–22. 10.1002/lary.21425 21287564 PMC3079336

[32] Langguth B Landgrebe M Frank E Schecklmann M Sand PG Vielsmeier V. Efficacy of different protocols of transcranial magnetic stimulation for the treatment of tinnitus: Pooled analysis of two randomized controlled studies. World J Biol Psychiatry. (2014) 15:276–85. 10.3109/15622975.2012.708438 22909265

[33] Brunoni AR Ferrucci R Bortolomasi M Scelzo E Boggio PS Fregni F. Interactions between transcranial direct current stimulation (tDCS) and pharmacological interventions in the Major Depressive Episode: findings from a naturalistic study. Eur Psychiatry. (2013) 28:356–61. 10.1016/j.eurpsy.2012.09.001 23182847

[34] Brunoni AR Valiengo L Baccaro A Zanao TA de Oliveira JF Goulart A. The sertraline vs. electrical current therapy for treating depression clinical study: results from a factorial, randomized, controlled trial. JAMA Psychiatry. (2013) 70:383–91. 10.1001/2013.jamapsychiatry.32 23389323

[35] Brunoni AR Junior RF Kemp AH Lotufo PA Bensenor IM Fregni F. Differential improvement in depressive symptoms for tDCS alone and combined with pharmacotherapy: an exploratory analysis from the sertraline vs. electrical current therapy for treating depression clinical study. Int J Neuropsychopharmacol. (2014) 17:53–61. 10.1017/S1461145713001065 24060107

[36] Vanneste S van der Loo E Plazier M De Ridder D. Parietal double-cone coil stimulation in tinnitus. Exp Brain Res. (2012) 221:337–43. 10.1007/s00221-012-3176-7 22782482

[37] Langguth B Zowe M Landgrebe M Sand P Kleinjung T Binder H. Transcranial magnetic stimulation for the treatment of tinnitus: a new coil positioning method and first results. Brain Topogr. (2006) 18:241–7. 10.1007/s10548-006-0002-1 16845596

[38] Song JJ Kim K Sunwoo W Mertens G Van de Heyning P De Ridder D. A Quantitative electroencephalography study on cochlear implant-induced cortical changes in single-sided deafness with tinnitus. Front Hum Neurosci. (2017) 11:210. 10.3389/fnhum.2017.00210 28572760 PMC5435818

[39] Lee SY Rhee J Shim YJ Kim Y Koo JW De Ridder D. Changes in the resting-state cortical oscillatory activity 6 months after modified tinnitus retraining therapy. Front Neurosci. (2019) 13:1123. 10.3389/fnins.2019.01123 31680845 PMC6813998

[40] McCombe A Baguley D Coles R McKenna L McKinney C Windle-Taylor P. Guidelines for the grading of tinnitus severity: the results of a working group commissioned by the British Association of Otolaryngologists, Head and Neck Surgeons, 1999. Clin Otolaryngol Allied Sci. (2001) 26:388–93. 10.1046/j.1365-2273.2001.00490.x 11678946

[41] Shore S Zhou J Koehler S. Neural mechanisms underlying somatic tinnitus. In: Moller A Langguth B Hajak G Kleinjung T Cacace A editors. Tinnitus: Pathophysiology and Treatment. Amsterdam: Elsevier (2007). p. 107–548. 10.1016/S0079-6123(07)66010-5

[42] Dehmel S Pradhan S Koehler S Bledsoe S Shore S. Noise overexposure alters long-term somatosensory-auditory processing in the dorsal cochlear nucleus–possible basis for tinnitus-related hyperactivity? J Neurosci. (2012) 32:1660–71. 10.1523/JNEUROSCI.4608-11.2012 22302808 PMC3567464

[43] Chen YC Wang F Wang J Bo F Xia W Gu JP. Resting-State Brain Abnormalities in Chronic Subjective Tinnitus: A Meta-Analysis. Front Hum Neurosci. (2017) 11:22. 10.3389/fnhum.2017.00022 28174532 PMC5258692

[44] Chen YC Liu S Lv H Bo F Feng Y Chen H. Abnormal resting-state functional connectivity of the anterior cingulate cortex in unilateral chronic tinnitus patients. Front Neurosci. (2018) 12:9. 10.3389/fnins.2018.00009 29410609 PMC5787069

[45] Zigmond AS Snaith RP. The hospital anxiety and depression scale. Acta Psychiatr Scand. (1983) 67:361–70. 10.1111/j.1600-0447.1983.tb09716.x 6880820

[46] Kreuzer PM Poeppl TB Bulla J Schlee W Lehner A Langguth B. A proof-of-concept study on the combination of repetitive transcranial magnetic stimulation and relaxation techniques in chronic tinnitus. J Neural Transm. (2016) 123:1147–57. 10.1007/s00702-016-1588-4 27315823

[47] Kreuzer PM Poeppl TB Rupprecht R Vielsmeier V Lehner A Langguth B. Individualized repetitive transcranial magnetic stimulation treatment in chronic tinnitus? Front Neurol. (2017) 8:126. 10.3389/fneur.2017.00126 28428769 PMC5382205

[48] Landgrebe M Hajak G Wolf S Padberg F Klupp P Fallgatter AJ. 1-Hz rTMS in the treatment of tinnitus: a sham-controlled, randomized multicenter trial. Brain Stimul. (2017) 10:1112–20. 10.1016/j.brs.2017.08.001 28807845

